# Clinical significance of the correlation between PLCE 1 and PRKCA in esophageal inflammation and esophageal carcinoma

**DOI:** 10.18632/oncotarget.16635

**Published:** 2017-03-28

**Authors:** Yongchen Guo, Yonghua Bao, Ming Ma, Shanshan Zhang, Yongmeng Zhang, Ming Yuan, Bing Liu, Yiqiong Yang, Wen Cui, Emmanuel Ansong, Huali Dong, Virgilia Macias, Wancai Yang

**Affiliations:** ^1^ Department of Pathology and Institute of Precision Medicine, Jining Medical University, Jining 272067, China; ^2^ Department of Thoracic Surgery, Affiliated Hospital, Jining Medical University, Jining 272067, China; ^3^ Department of Pathology, Xinxiang Medical University, Xinxiang 453003, China; ^4^ Department of Pathology, University of Illinois at Chicago, Chicago, IL 60612, USA

**Keywords:** esophagitis, Barrett's esophagus, squamous cell carcinoma, adenocarcinoma, PLCE1

## Abstract

Esophagitis and Barrett's esophagus are linked to esophageal squamous cell carcinoma and adenocarcinoma, respectively. However, the underlying mechanisms are still unclear. This study analyzed the expression levels of and correlation between PLCE1 and PRKCA in human esophagitis, carcinogen NMBA-induced rat esophagus, PLCE1 genetic deficient mouse esophageal epithelial tissues and human esophageal cancer cell line, integrated with Online oncology data sets. We found that the expression levels of both PLCE1 and PRKCA were significantly elevated in human esophagitis, esophageal squamous cell carcinoma, Barrett's esophagus, esophageal adenocarcinoma and in NMBA-treated rat esophageal epithelia. However, PRKCA and cytokines were significantly downregulated in PLCE1-deficient mouse esophageal epithelia, and knockdown of PLCE1 in human esophageal cancer cells led to reduction of PRKCA and cytokines. Finally, high expression of both PLCE1 and PRKCA is significantly associated with poor outcomes of the patients with esophageal cancers. In conclusion, this study defined the initiation and progression of esophageal inflammation and malignant transformation, in which the positive correlation of PLCE1 and PRKCA exhibits critical clinical significance.

## INTRODUCTION

Esophageal cancer is one of the most common cancer types worldwide and is frequently fatal; 5-year survival is approximately 13% [1]. The two major histological subtypes - squamous cell carcinoma (ESCC) and adenocarcinoma (EAC) differ substantially in their underlying patterns of incidence and key etiologic factors. For instance, ESCC has a striking, non-random, geographic distribution worldwide with higher prevalence in certain areas of northern China, central Asia and southern Africa [1]. In the United States, ESCC develops more often in African-American males and has slightly declined in incidence, but the incidence of EAC has risen 10-fold over the past three decades for reasons not well understood [2]. The exact causes and underlying mechanisms by which these cancers develop remain unknown. However, epidemiological studies have provided clues regarding the interaction of environmental and genetic factors in esophageal cancer development [3–5]. Recent studies have also shown that ESCC and EAC are attributable to esophageal stress or chronic inflammation, resulted from nutritional deficiencies, intake of nitrosamine-rich or mycotoxin-contaminated foods, drinking of extremely hot beverages and low socioeconomic status [6]. The development of ESCC in Europe and North America has been linked to heavy smoking and alcohol consumption [7–9]. EAC is mainly originated from Barrett's esophagus, a pre-malignant condition in which normal squamous epithelium is replaced by metaplastic columnar epithelium [10]. The causes of the increase of Barrett's esophagus and EAC could be diet and obesity. The gut microbiota is strongly associated with obesity and has been shown to aid in the pathogenesis of obesity-associated gastrointestinal cancers, such as colorectal cancer, through regulation of inflammatory processes [11]. However, how esophageal inflammation develops and the link between inflammation and esophageal cancer is not well characterized.

Phospholipase C epsilon 1 (PLCE 1, or PLCE1) is one of members of the phosphoinositide-specific enzyme phospholipase C that converts phosphatidylinositol 4,5-biphosphate (PtdIns (4, 5) P2) to two intracellular second messengers, diacylglycerol and inositol 1,4,5-trisphosphate, regulates protein kinase C (PRKC) activity and calcium mobilization, respectively [12–15], which are physiologically involved in the maturation of cell growth and differentiation and are pathologically involved in inflammation development, progression and carcinogenesis [16]. Previous studies have shown that PLCE1 regulates inflammatory cytokine production, and thus, participates in skin and colorectal cancer formation [17–19]. The studies from us and others have revealed the risk susceptibility of the single nucleotide polymorphism (SNP, rs2274223: A5780G:His1927Arg) of PLCE1 in esophageal carcinoma [20–23], we have further shown that the 5780G allele in PLCE participates in the inflammatory process in esophageal epithelium and is associated with esophagitis and esophageal cancer [24]. However, whether the expression of PLCE1 and PRKC is associated with esophageal inflammation and progression to esophageal cancer is not clear.

Through deeply mining the oncology database and determining the expression of PLCE1 and PRKCA in human esophageal biopsies, esophageal cancer tissue microarray, carcinogen NMBA-induced rat model and specific PLCE1 knockout mouse model, we have found that the expression of PLCE1 and PRKCA are elevated during esophageal inflammation development, progression and malignant transformation. The positive correlation between PLCE1 and PRKCA might have important clinical significance.

## RESULTS

### mRNA expression levels of PLCE1 and PRKCA were increased in esophagitis and esophageal squamous cell carcinoma (ESCC)

**Figure 1:**
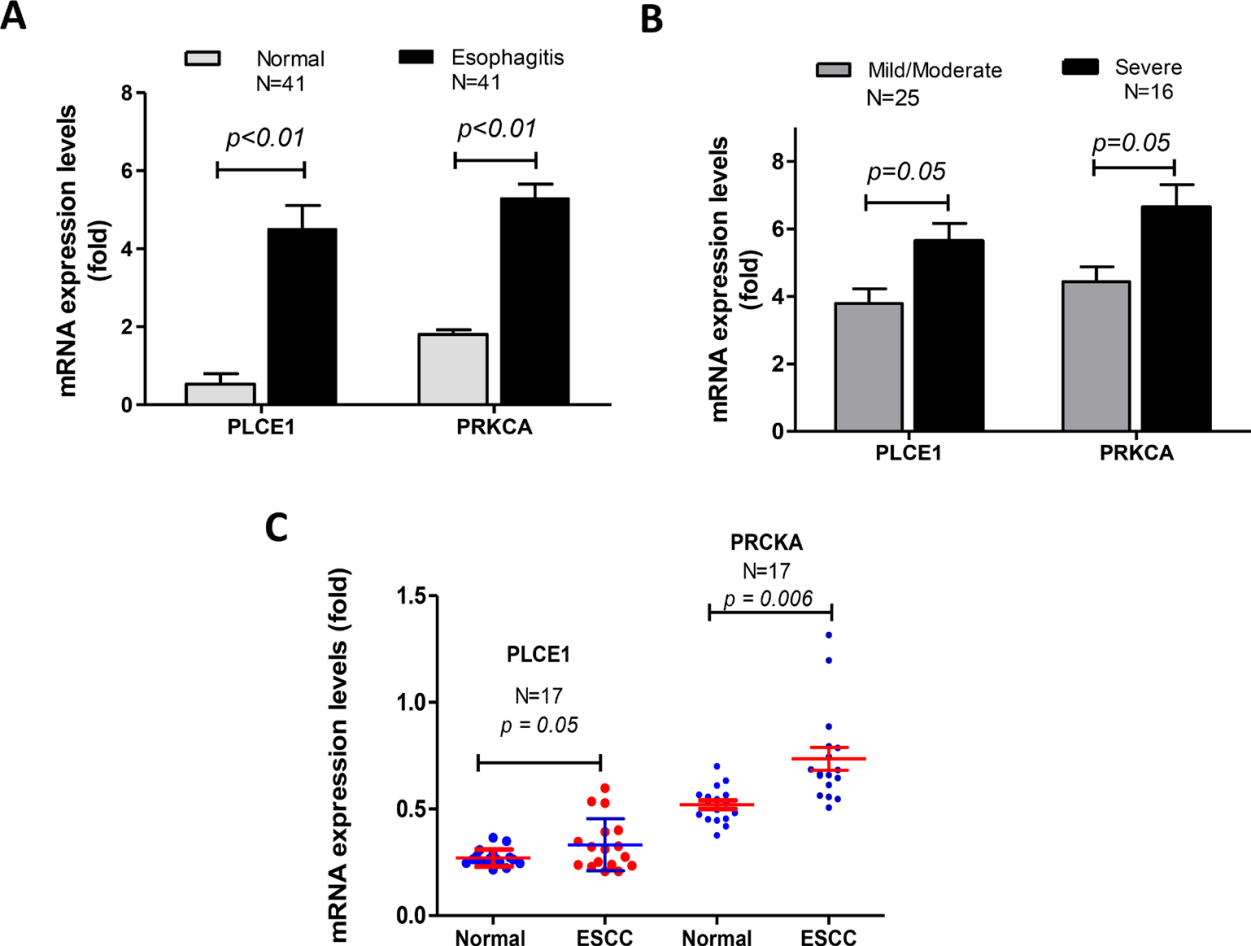
mRNA levels of PLCE1 and PRKCA were significantly increased in esophagitis and esophageal squamous cell carcinoma (ESCC) (**A**) PLCE1 and PRKCA mRNA levels were increased in esophagitis, in comparison with their matched normal mucosa. (N, the number of patients.) (**B**) PLCE1 and PRKCA mRNA was significantly elevated in severe esophagitis tissues than those in the mild or moderate esophagitis. (N, the number of patients in each group.) (**C**) PLCE1 and PRKCA mRNA levels were increased in ESCC, compared to the adjacent normal esophageal mucosa (17 patients in each group).

We have previously reported that PLCE1 single nucleotide polymorphism (SNP rs2274223: A5780G:His1927Arg) was linked to esophagitis [24], but it was not clear whether PLCE1 expression levels are associated with esophagitis. Thus, we conducted quantitative RT-PCR in 41 pairs of esophageal biopsies of esophagitis and their adjacent normal mucosa. We found that PLCE1 mRNA expression levels were significantly increased in the esophagitis, compared to the normal esophageal mucosa (Figure 1A). Similar changes of PRKCA were also observed (Figure 1A). We then classified the 41 esophagitis into 25 mild /moderate and 16 severe esophagitis on the basis of disease pathology degree. Interestingly, we found that PLCE1 and PRKCA mRNA levels were even more elevated in severe esophagitis, compared to mild/moderate esophagitis (Figure 1B).

We have previously reported that PLCE1 mRNA levels were increased in esophageal squamous cell carcinoma (ESCC), which was supported by published data base Oncomine, reported by Hu et al [25]. We deeply mined the data and found that in the 17 pairs of ESCC, PLCE1 mRNA levels were slightly increased, compared to the adjacent normal esophageal mucosa (*p* = 0.05), and PRKCA mRNA levels were dramatically elevated in ESCC (*p* = 0.006, Figure 1C).

### mRNA expression levels of PLCE1 and PRKCA were increased in Barrett's Esophagus(BE) and esophageal adenocarcinoma (EAC)

**Figure 2:**
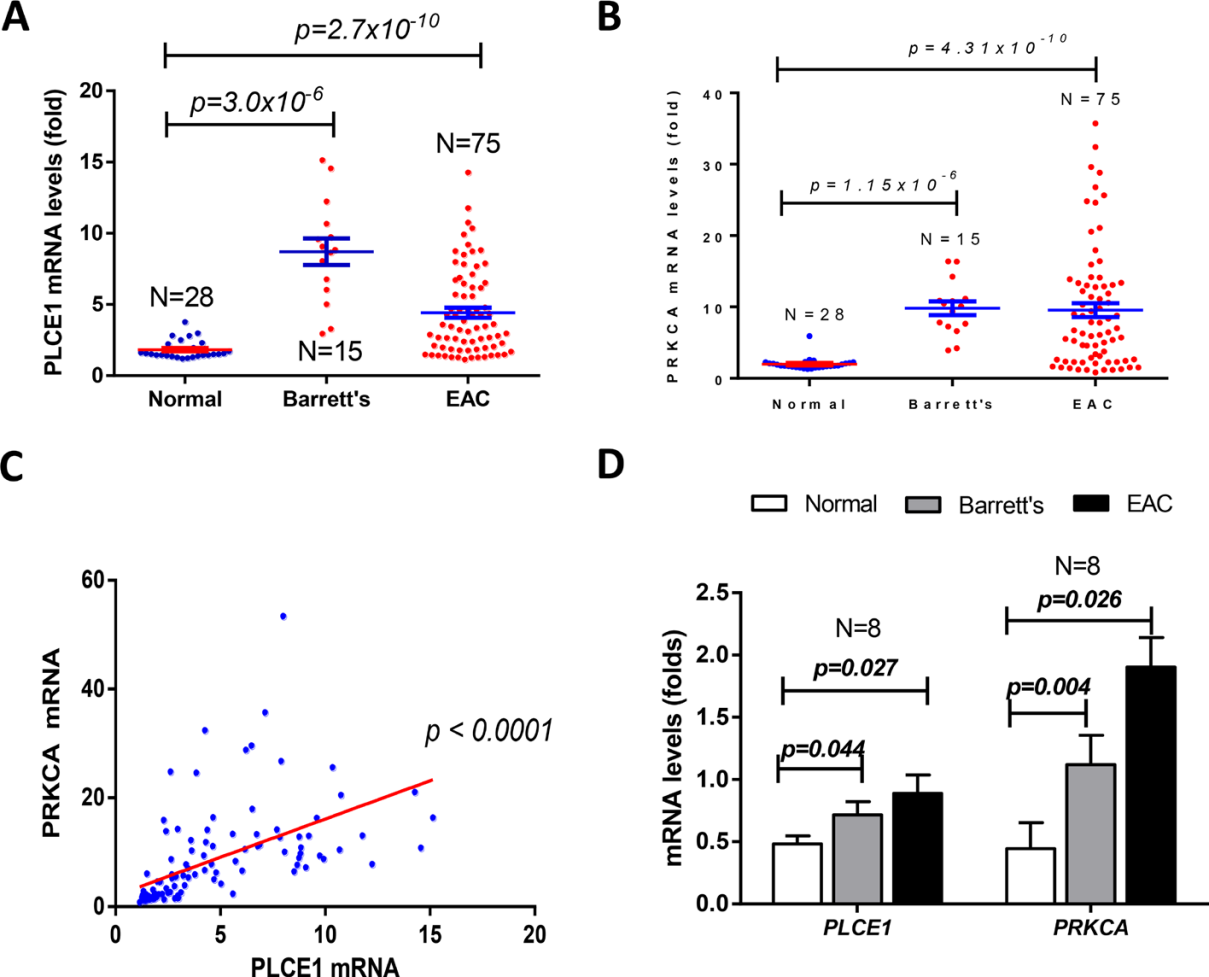
mRNA levels of PLCE1 and PRKCA were significantly increased in Barrett's Esophagus (BE) and esophageal adenocarcinoma (EAC) (**A**) PLCE1 mRNA levels were increased in Barrett's Esophagus and EAC; (**B**) PRKCA mRNA levels were increased in Barrett's Esophagus and EAC; (**C**) the mRNA levels of PLCE1 and PRKCA were positively correlated; (**D**) another set of BE and EAC data showed the similar changes of mRNA levels of PLCE1 and PRKCA in BE and EAC.

Barrett's Esophagus is one of the most esophageal inflammations in the Western world and is one of the major causes of esophageal adenocarcinoma. To investigate the changes of PLCE1 and PRKCA in BE and EAC, we deeply mined the Kim's Oncomine data [26] and found that both PLCE1 and PRKCA mRNA levels were dramatically increased in Barrett's Esophagus and EAC, in comparison with the non-tumor esophageal mucosa (Figure 2A and 2B). Moreover, the expression levels of PLCE1 and PRKCA were positively correlated (Figure 2C). Similar results were observed (Figure 2D) by deeply mining another set of Barrett's Esophagus and EAC data, reported by Kimchi et al. [27].

### PLCE1 and PRKCA mRNA levels were increased in NMBA-treated rat esophageal mucosa

**Figure 3:**
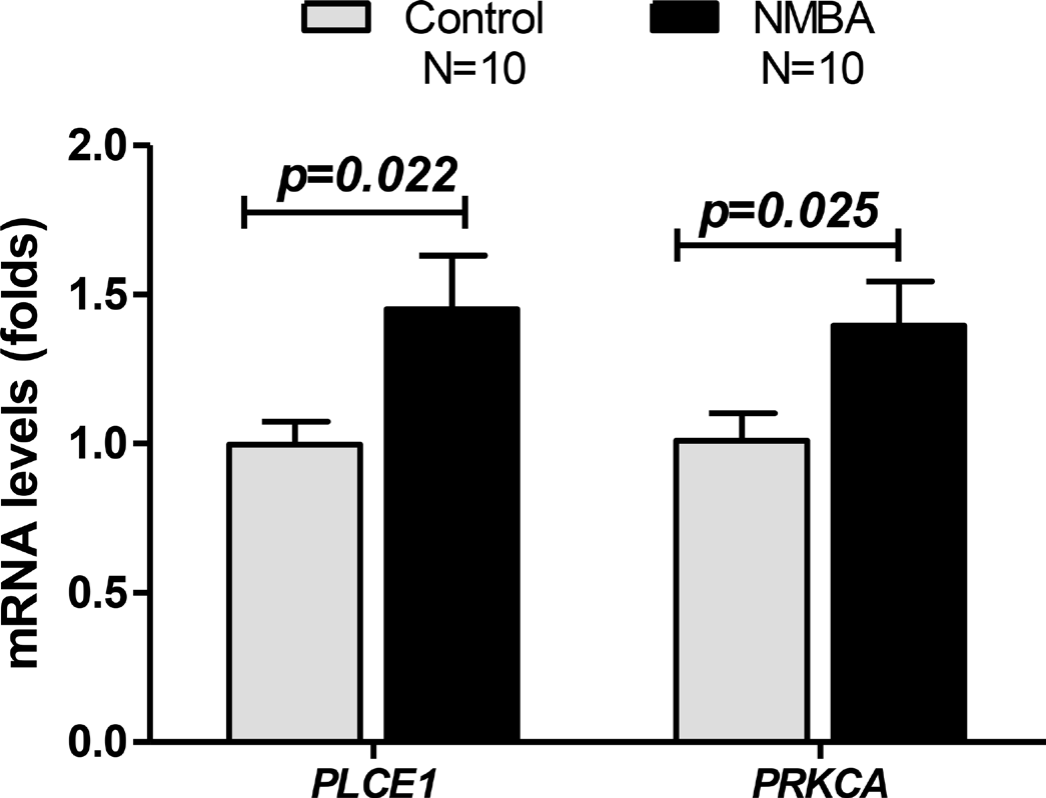
PLCE1 and PRKCA mRNA levels were increased in N-nitrosomethylbenzylamine (NMBA)-treated rat esophageal mucosa Rat esophageal mucosa was isolated from formalin-fixed paraffin-embedded sections, total RNA was extracted using Roche High Pure FFPE kit, mRNA levels of PLCE1 and PRKCA were measured by qRT-PCR. 10 rats from each group of NMBA-treated or no treated rats were studied.

Epidemiology and clinical studies have demonstrated that nitrite is a carcinogen for esophagitis and esophageal cancer, and thus, NMBA has been widely used to induce rodent models of esophageal inflammation and cancer. To determine the alterations of PLCE1 and PRKCA in NMBA-induced rat esophageal lesions, we detected rat esophagus from NMBA-treated rat model provided by Dr Li-Shu Wang [28] (Medical College of Wisconsin, Milwaukee, WI). Rat esophageal epithelial cells were scraped from formalin-fixed paraffin-embedded slides, total RNA was extracted and qRT-PCR was performed. We found that both PLCE1 and PRKCA mRNA levels were also significantly increased in NMBA-treated rat esophageal epithelial cells, compared to the control rats without NMBA treatment (Figure 3).

### Cytokines and PRKCA mRNA levels were significantly reduced in PLCE1 knockout mouse esophagus

**Figure 4:**
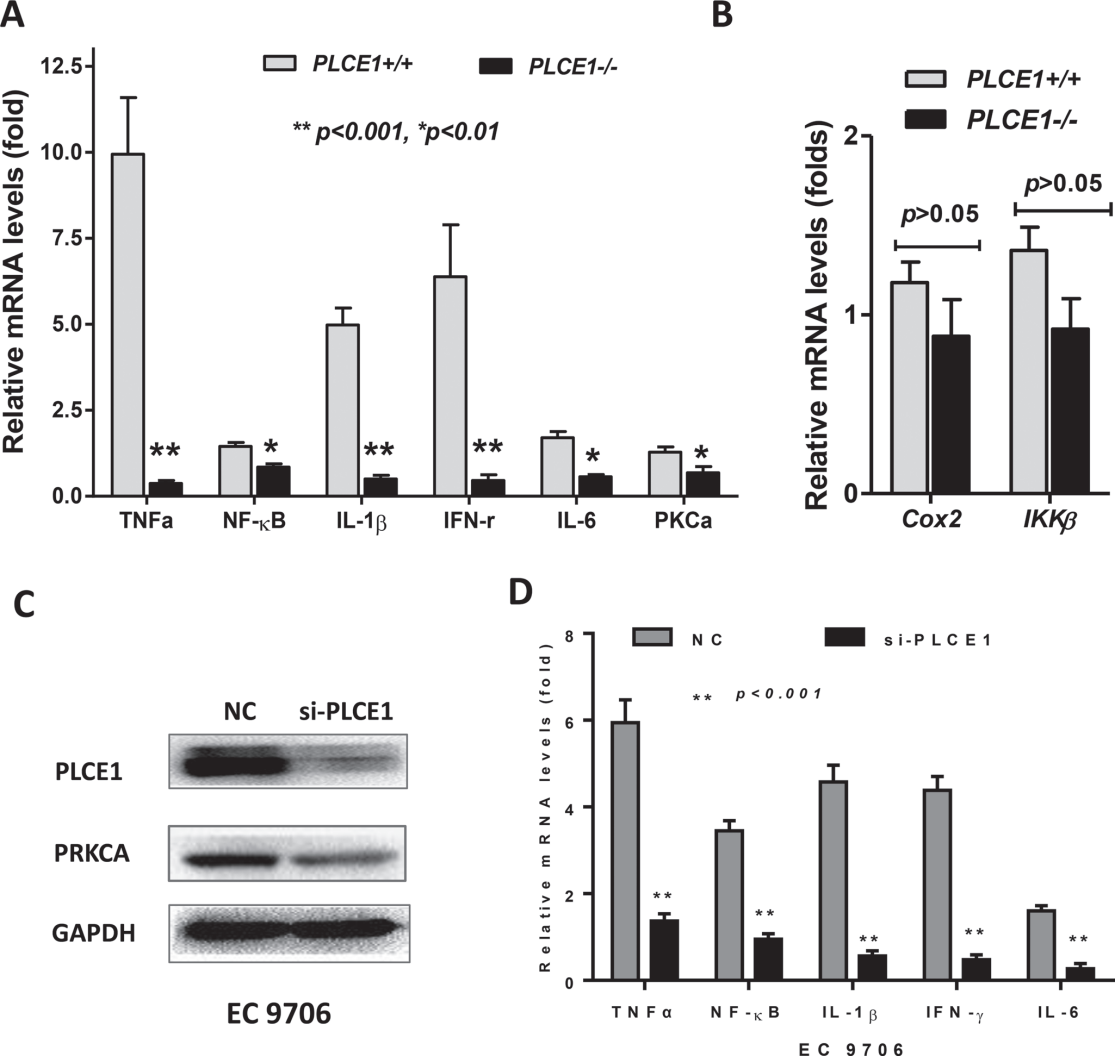
The expression of PRKCA and cytokines was reduced in PLCE1 knockout mouse esophagus and in PLCE1-reduced human ESCC cell line EC9706 (**A**) significantly changed cytokines in mouse esophageal epithelia, assayed by qRT-PCR; (**B**) Cox2 and IKKβ were also reduced in mouse esophagus; (**C**) PLCE1 and PRKCA were reduced by siRNA targeting human PLCE1 in EC9706 cells, by western blotting; (**D**) cytokines were reduced in EC9706 cells resulted by PLCE1 knockdown. *PLCE1 +/+*, *PLCE1* wild-type mouse esophageal mucosa; *PLCE1−/−, PLCE1* knockout mouse esophageal mucosa. *p < 0.01, **p < 0.001, in comparison between *PLCE1+/+* and *PLCE1−/−* mouse esophageal mucosa. NC (negative control), scramble siRNA; si-PLCE1, siRNA targeting human PLCE1.

Since PLCE1 and PRKCA were increased in esophageal inflammation (esophagitis and Barrett's Esophagus), we then determined whether PLCE1 was linked to esophageal inflammation, we analyzed mouse esophageal mucosa isolated from the PLCE1 gene knockout mouse models (PLCE1−/−) provided by Dr. Alan Smrcka from University of Rochester (Rochester, NY) [29]. Compared to the wild-type mouse esophagus, the cytokines (e.g. TNFα, NF-kB, IL-1β, IFN-γ, IL-6) and PRKCA mRNA levels were significantly decreased in PLCE1−/− mouse esophagus (Figure 4A). In addition, Cox2 and IKKβ were also decreased in PLCE1−/− mouse esophagus, although the statistically analysis were not significant (Figure 4B).

### Knockdown of PLCE1 led to reduction of PRKCA and cytokines in human esophageal cancer cells

To confirm the correlation between PLCE1 and PRKCA *in vitro*, we used siRNA targeting human PLCE1 in esophageal squamous cell carcinoma cell line EC9706 cells and found that PRKCA protein expression was downregulated once PLCE1 was knocked down (Figure 4C), compared to the scramble control group (negative control, NC), assayed by western blotting. In addition, qRT-PCR showed that human cytokines in cultured EC9706 cells were also significantly decreased (Figure 4D), similar changes as seen in the PLCE1 −/− mouse esophagus.

### Differential expression of PLCE1 and PRKCA and their association with clinicopathological characteristics of ESCC

**Figure 5:**
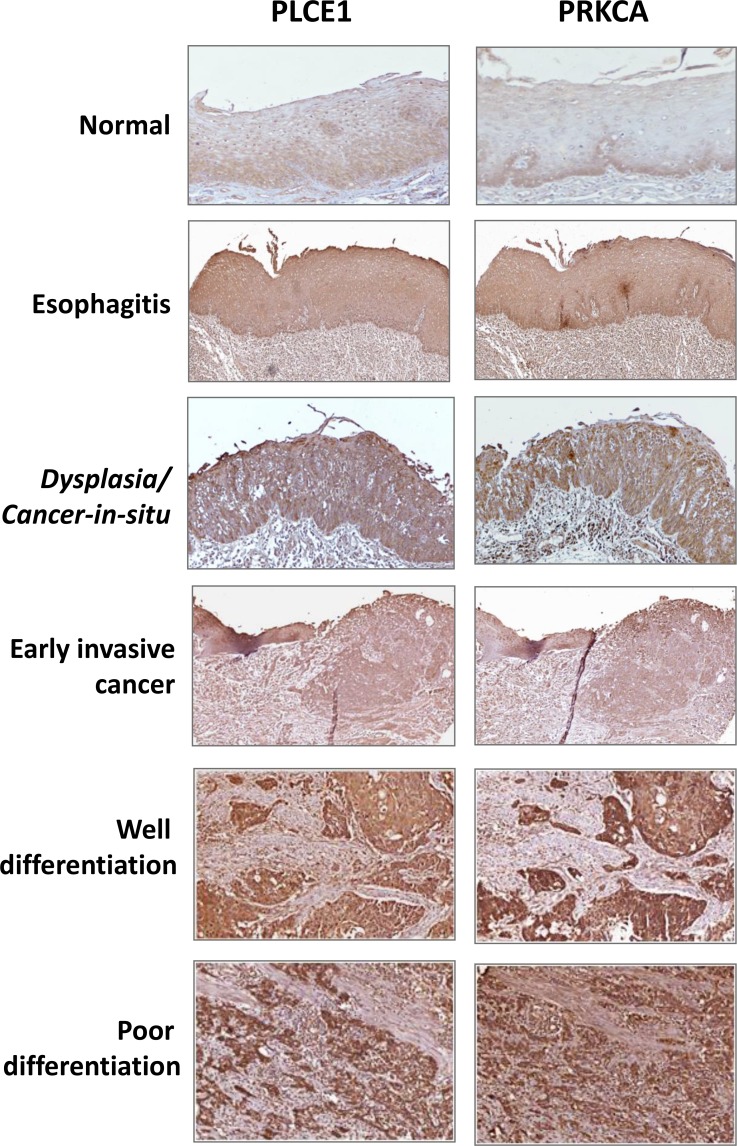
The protein expression level of PLCE1 and PRKCA in normal esophageal tissues and ESCC The expression of PLCE1 and PRKCA were gradually increased from normal esophagus to cancer-in-situ, well differentiation and poorly differentiation ESCC.

We have previously reported that PLCE1 expression levels were increased in limited number of ESCC samples [24], herein, we used an ESCC tissues microarray (TMA) that contained 362 cases of esophageal squamous cell carcinoma with complete clinical information and follow-up results, including some cancer-in-situ and early invasive ESCC, and matched normal mucosa, esophagitis, and conducted immunohistochemical staining with anti-human PLCE1 and PRKCA antibodies, respectively. As reported previously and as shown in Figure 5, PLCE1 was slightly expressed in normal esophageal epithelial cells, mainly located at the basal layers and proliferation zone, but the expression was increased in cancer-in-situ, and was highly expressed in ESCC, further, poor-differentiated ESCC showed strongest staining of PLCE1 staining, PRKCA staining was also gradually increased with the progression of ESCC. Immunohistochemical staining scores and statistical analysis showed that PLCE1 expression levels were significantly associated with tumor differentiation (Table 1), that was, 69% of well-differentiated ESCC showed strong staining of PLCE1 (Score 3 or 2), and 31% of well-differentiated ESCC showed weak or no staining (Score 1 or 0), but 79% of poorly-differentiated ESCC showed strong staining of PLCE1 (Score 3 or 2), and 21% of poorly-differentiated ESCC showed weak or no staining (Score 1 or 0) (*p* = 0.038). In addition, 50% of cancer *in situ* showed strong staining, but 73% of invasive ESCC showed strong staining of PLCE1 (*p* = 0.032). Interestingly, ESCC with lymphocyte infiltration, meaning chronic esophageal inflammation, showed stronger PLCE1 staining, compared to the ESCC without lymphocyte infiltration (*p* = 0.011). Similar expression patterns and scores were observed with PRKCA staining in the ESCC TMA, demonstrating a positive correlation between PLCE1 and PRKCA.

**Table 1:** Associations of PLCε1 or PKCα protein expression with clinicopathological characteristics of 362 esophageal squamous cell carcinoma (ESCC) patients

Clinicopathologic	*N*	PLCε1 expression			PKCα expression		
Variable		3 and 2	1 and 0	*P* value	3 and 2	1 and 0	*P* value
**Age**				0.651			0.116
≤ 60	158	112 (71%)	46 (29%)		87 (55%)	71 (45%)	
> 60	204	149(73%)	55 (27%)		129 (63%)	75 (27%)	
**Gender**				0.133			0.695
Male	230	172(75%)	58 (25%)		139(60%)	91 (40%)	
Female	132	89 (67%)	43(33%)		77 (58%)	55 (42%)	
**Smoking**				0.075			0.872
Yes	154	119 (77%)	35(23%)		92 (60%)	62 (40%)	
No	208	142 (68%)	66(32%)		126 (61%)	82 (39%)	
**Drinking**				0.122			0.079
Yes	100	78 (78%)	22(22%)		67 (67%)	33 (33%)	
No	262	183 (70%)	79 (30%)		149 (57%)	113(43%)	
**Lymphatic metastasis**				0.491			0.989
Yes	161	119 (74%)	42 (26%)		96 (60%)	65 (40%)	
No	201	142 (71%)	59 (29%)		120 (60%)	81 (40%)	
**Lymphocyte infiltration**				***0.011***			***0.013***
Yes	207	160(77%)	47(23%)		135 (65%)	72 (35%)	
No	155	101(65%)	54(35%)		81 (52%)	74 (48%)	
**Tumor differentiation**				***0.038***			***0.006***
Well/moderate	235	161(69%)	74 (31%)		128(54%)	107(46%)	
Poor	127	100 (79%)	27 (21%)		88(69%)	39(31%)	
				***0.032***			***0.005***
*Cancer in situ*	18	9(50%)	9(50%)		5(28%)	13 (72%)	
Invasive cancer	344	252 (73%)	92 (27%)		211 (61%)	133 (39%)	

Although the expression levels of PLCE1 and PRKCA were not associated with age, and gender, but PLCE1 expression levels were slightly associated with smoking (smoking patients showed stronger PLCE1, *p* = 0.075), and PRKCA expression levels were slightly associated with drinking (drinkers showed stronger PRKCA staining, *p* = 0.079).

### The association between the expression of PLCE1 and PRKCA and ESCC patient survival time

**Figure 6:**
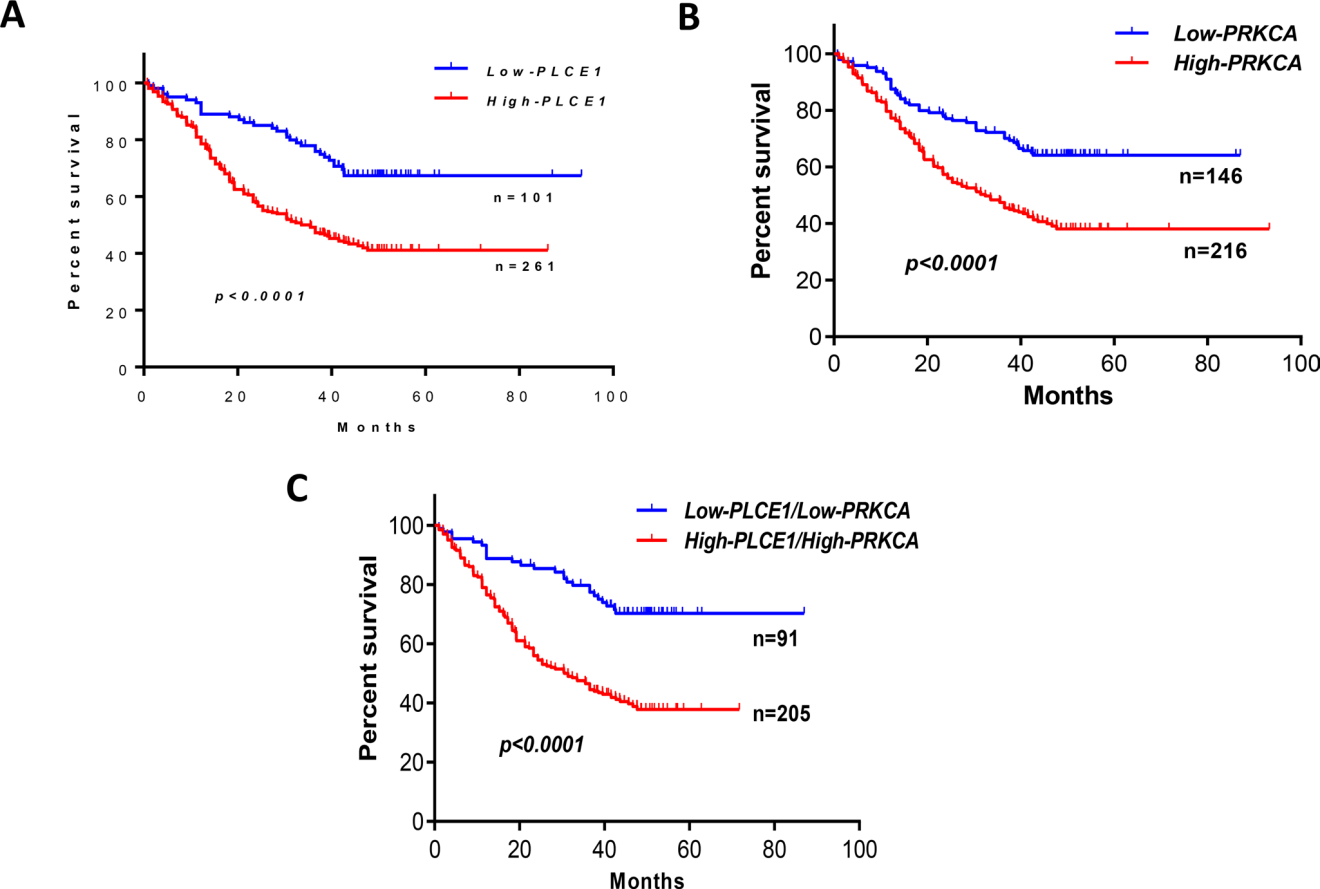
Survival curves for 362 esophageal squamous cell carcinoma (ESCC) patients according to expression patterns of PLCE1 and PRKCA protein in tumor tissues Kaplan-Meier analysis showed that the survival time of ESCC patients with high PLCE1 expression or high PRKCA mRNA expression were markedly shorter than that with low PLCE1 expression or low PRKCA mRNA expression (both *p* < 0.0001; (**A** and **B**). But, high expression of both PLCE1 and PRKCA showed the worst outcomes, in comparison with the low expression of both PLCE1 and PRKCA (*p* < 0.0001; (**C**).

Since the expression of PLCE1 and PRKCA was associated with clinicopathological characteristics, we then determined whether the expression of PLCE1 and PRKCA is linked to ESCC patient survival time. As shown in Figure 6A, higher expression of PLCE1 in ESCC was associated with poor survival, compared to the patients with lower expression of PLCE1 (*p* < 0.0001), regardless the expression levels of PRKCA. Moreover, higher expression of PRKCA in ESCC was associated with shorter survival, compared to the patients with lower expression of PRKCA (*p* < 0.0001), regardless the expression levels of PLCE1 (Figure 6B). Further, higher expression of both PLCE1 and PRKCA showed the worst outcomes, in comparison with the lower expression of both PLCE1 and PRKCA (Figure 6C, *p* < 0.0001).

### PLCE1 and PRKCA mRNA levels and their association were also observed in other cancers

**Figure 7:**
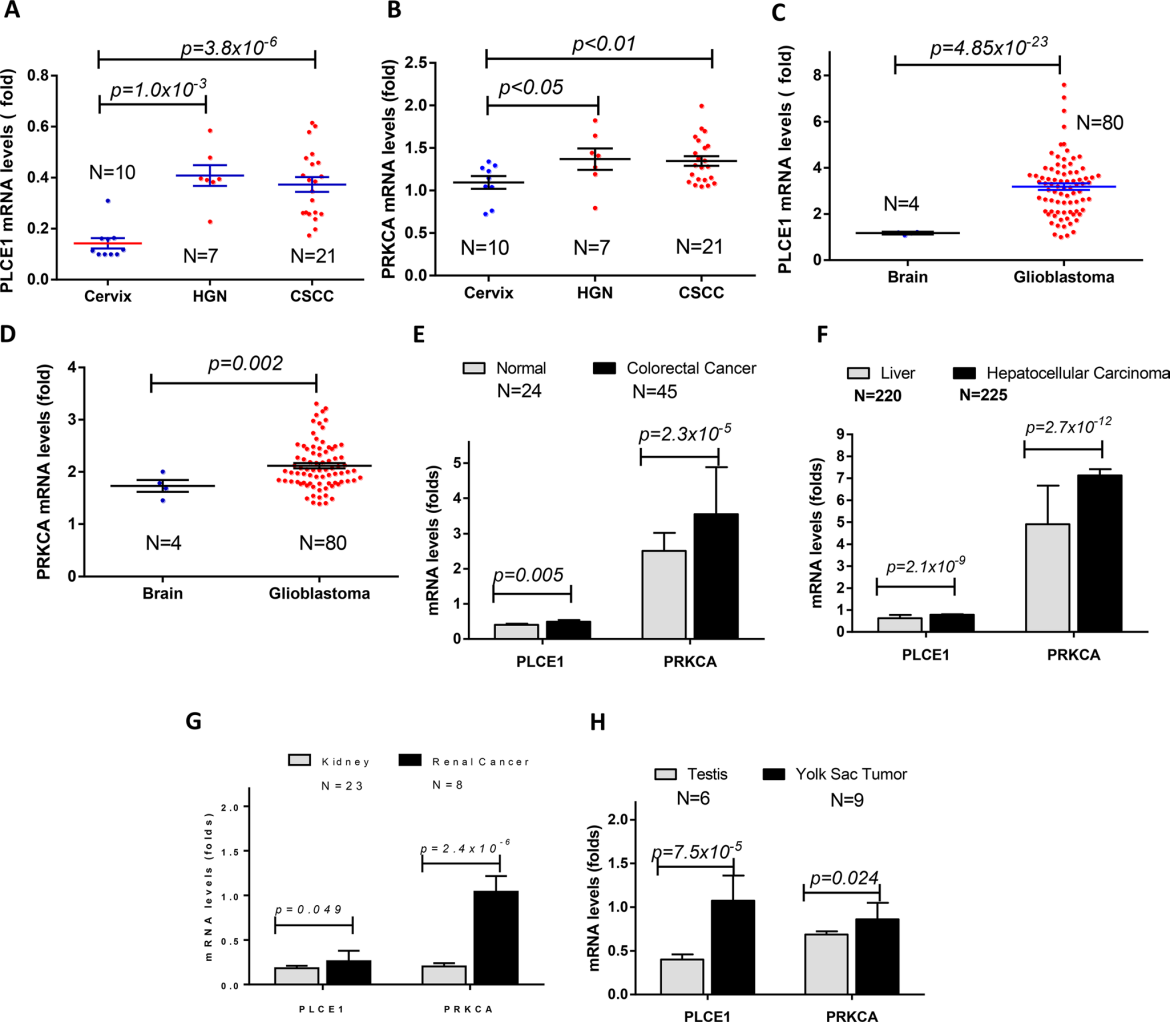
The expression levels of PLCE1 and PRKCA in other cancers PLCE1 and PRKCA mRNA levels were elevated in human high grade introepithelial neoplasm (HGN) and cervical squamous cell carcinoma (CSCC) (**A** and **B**), brain glioblastoma (**C** and **D**), colorectal cancer (**E**), hepatocellular carcinoma (**F**), renal cancer (**G**) and Yolk Sac tumors (**H**).

To confirm the correlation of PLCE1and PRKCA in other cancers, we integrated and deeply mined online Oncomine data sets (https://www.oncomine.com). Cervical cancer data was extracted from Zhai Cervix [30], glioblastoma was extracted from Murat Brain Glioblastoma [31], colorectal cancer data was extracted from Skrzypczak Colorectal Cancer [32], hepatocellular carcinoma data was extracted from Roessler Liver Cancer [33], renal cancer data was extracted from Jones Renal Cancer [34], and yolk sac tumor data was extracted from Korkola Seminoma [35]. The results showed that the expression levels of both PLCE1 and PRKCA were also elevated in above cancers. For instance, PLCE1 was significantly increased from 0.14 ± 0.06 in normal cervix (*n* = 10) to 0.41 ± 0.11 in cervical high grade introepithelial neoplasm (HGN, *n* = 7) and 0.37 ± 0.15 in cervical squamous cell carcinoma (*n* = 21)(Figure 7A). PRKCA was significantly increased from 1.09 ± 0.22 in normal cervix (*n* = 10) to 1.35 ± 0.47 in cervical high grade introepithelial neoplasm (HGN, *n* = 7) and 1.37 ± 0.08 in cervical squamous cell carcinoma (*n* = 21) (Figure 7B). In brain glioblastoma, PLCE1 was increased from 1.17 ± 0.06 in normal (*n* = 4) to 3.18 ± 1.28 in glioblastoma (*n* = 80) (Figure 7C), and PRKCA was increased from 1.73 ± 0.23 (*n* = 4) in normal to 2.11 ± 0.44 in glioblastoma (*n* = 80) (Figure 7D). In colorectal cancer, PLCE1 was slightly increased from 0.40 ± 0.03 in normal mucosa (*n* = 24) to 0.49 ± 0.04 in colorectal cancer (*n* = 45) (Figure 7E), and PRKCA was increased from 2.50 ± 0.51 in normal mucosa to 3.54 ± 1.34 in glioblastoma (Figure 7E). In hepatocellular carcinoma, PLCE1 was significantly increased from 0.62 ± 0.15 in normal (*n* = 220) to 0.78 ± 0.34 in hepatocellular carcinoma (*n* = 225) (Figure 7F), and PRKCA was increased from 4.90 ± 1.46 in normal to 7.13 ± 4.22 in hepatocellular carcinoma (Figure 7F). The significant increase of PLCE1 and PRKCA was also observed in renal cancer (Figure 7G) and Yolk Sac Seminoma (Figure 7H). All of the changes supported the results that were observed in esophageal inflammation and esophageal squamous cell carcinoma and adenocarcinoma.

## DISCUSSION

Using human esophageal samples, carcinogen NMBA-induced esophageal cancer rat model, genetic deficient mouse model, cultured cell, and deeply mining oncology data base, we have demonstrated that PLCE 1 and PRKCA are altered in a similar pattern during the development of esophageal inflammation (esophagitis and Barrett's esophagus) and malignant transformation (squamous cell carcinoma and adenocarcinoma). These findings suggest crucial clinical significance of their correlation, and the co-expression of PLCE1 and PRKCA might also be a indicator for esophageal cancer patient survival.

Numerous studies from epidemiology, clinical trials and animal models have demonstrated that most carcinogenesis, particularly, gastrointestinal tumors, is associated with chronic inflammation. It has been well known that Barrett's esophagus is the major cause of esophageal adenocarcinoma, during the progression, cytokines play critical role, for instance, increased expression of IL-1β in mouse leads to esophageal inflammation and esophageal adenocarcinoma in IL-1β transgenic mouse model [36]. This study provided further evidence that phospholipase C epsilon 1 might also be an important element that was significantly increased in Barrett's esophagus and in EAC, and genetic deletion of PLCE1 resulted in significant downregulation of the expression of some key cytokines, such as NF- κB, IL-1β, IL-6, TNFα, IFN-γ. Interestingly, PRKCA was also altered with the changes of PLCE1, suggesting the important significance of their correlation in the development of esophagitis and Barrett's esophagus and their malignant transformation.

**Figure 8:**
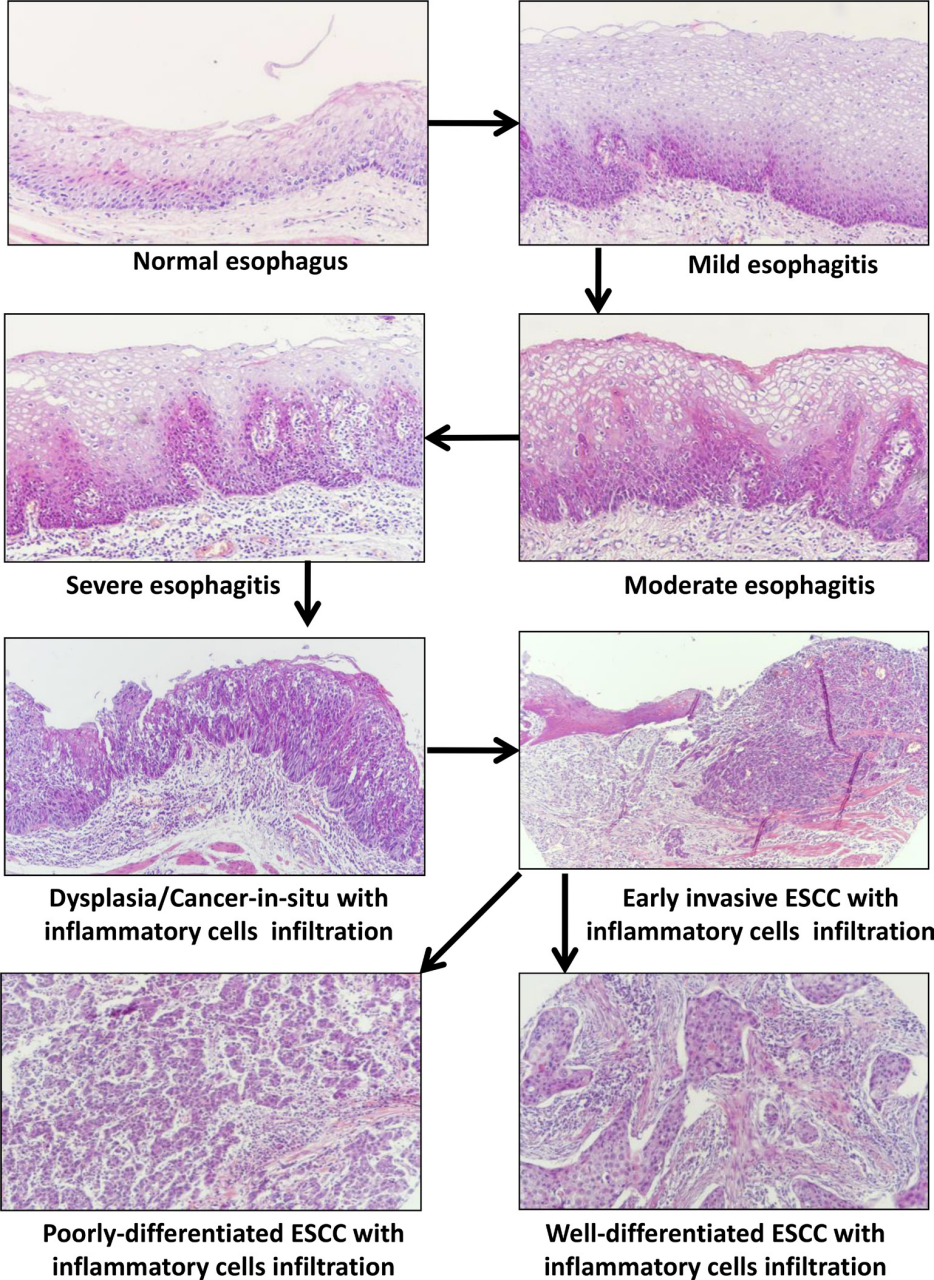
Schematic model of the development of esophagitis and its progression to esophageal squamous cell carcinoma (ESCC) This model demonstrates normal esophagus, esophagitis with mild, moderate or severe epithelial hyperplasia and inflammatory cells infiltration in sub-mucosa, epithelial dysplasia and cancer-in-situ with inflammatory cells infiltration, early ESCC and invasive cancer (well differentiation or poor differentiation with inflammatory cells infiltration).

The development of esophagitis and its progression from normal to mild, moderate and severe esophagitis, and finally to cancer *in situ* and invasive squamous cell carcinoma has not been well defined. Through this work with summarization of previous publications, we proposed the progression model of esophagitis development, progression and transformation in Figure 8, showing from normal esophagus to esophagitis with lymphocyte infiltration and hyperplasia, dysplasia and cancer-in-situ with lymphocyte infiltration, and finally to invasive cancer (well differentiation or poor differentiation) with lymphocyte infiltration. Importantly, we have demonstrated that PLCE1 and PRKCA are slightly expressed in normal esophageal epithelial cells, and that their expression levels are gradually increased during esophageal inflammation, progression, and malignant transformation – *cancer-in-situ* and invasive ESCC (Figure 5).

PLCE 1 is a phosphoinositide-specific enzyme phospholipase C that converts phosphatidylinositol 4,5-biphosphate (PtdIns (4, 5) P2) to two intracellular second messengers, diacylglycerol and inositol 1,4,5-trisphosphate, andregulate protein kinase C activity [12–16]. There are six members of the PLC family (PLCβ, γ, δ, ε, η and ζ) in mammalian cells and exert diverse functions in carcinogenesis, in terms of exhibiting distinct regulatory mechanisms although they share similar catalytic properties [37, 38]. PLCE binds to and is activated by the Ras family GTPases [12, 39, 40]. Previous studies have shown that PLCE1 regulates inflammatory cytokine production, and thus, participates in skin and colorectal cancer formation [17–19]. In the present work, we showed that the expression of PLCE1 was increased in Barrett's esophagus and inflammatory squamous epithelia, and significantly increased in esophageal adenocarcinoma and squamous cell carcinoma. Moreover, carcinogen NMBA-treated rat esophageal epithelial cells showed significant elevation of PLCE1, and in contrast, PLCE1 deficient mouse model exhibited decreased cytokines (i.e. decreased TNFα, NF-kB, IL-1β, IL-6, etc.). Above findings strongly indicate the important participation of PLCE1 in esophageal inflammation development and malignant transformation.

The upregulation of PLCE1 in esophageal inflammation and esophageal squamous cell carcinoma and adenocarcinoma was also observed in other types of cancers by deeply mined Oncomine data sets (https://www.oncomine.com), although the causes are still unclear. For example, cervical architecture and cervical squamous cell carcinogenesis is similar as esophagus and esophageal squamous cell carcinoma, and the upregulation of PLCE1 in high grade introepithelial neoplasm (HGN) and cervical squamous cell carcinoma (CSCC) was also similar as seen in esophageal cancer. As to hepatocellular carcinoma, the upregulation of PLCE1 could be caused by virus-stimulated inflammation because most hepatocellular carcinomas are linked to hepatitis virus.

Previous studies have revealed the risk susceptibility of PLCE1 polymorphism (rs2274223) in esophageal carcinoma [20–23], we have also shown that the 5780G allele in PLCE1 is associated with esophagitis and esophageal cancer in high-risk areas of ESCC [24]. This study provided additional evidence showing the importance of PLCE1 in esophageal inflammation development and carcinogenesis.

Protein kinase C is a family of calcium- and the second messenger diacylglycerol-activated and serine- and threonine-specific protein kinases, is involved in diverse cellular signaling pathways by phosphorylating a wide variety of protein targets [41]. PRKCA participates in and plays different roles in many cellular processes, such as cell proliferation, differentiation, apoptosis, cell adhesion and migration, cell transformation, tumorigenesis and inflammation, by activating signaling cascade (e.g. ERK1/2 and NF-kB) and by directly phosphorylating downstream targets (e.g. RAF1 and BCL2) [42, 43]. This study has demonstrated that PRKCA is involved in initiation and progression of esophageal epithelium inflammation, Barrett's esophagus, and carcinogenesis, in terms of significantly increased expression along with PLCE1 and cytokines in esophagitis, Barrett's esophagus, esophageal squamous cell carcinoma and adenocarcinoma, and in NMBA-treated rat esophageal epithelia, and it was decreased in mouse esophageal epithelia of PLCE1-deficient mouse model. Based on the model of esophagitis initiation and progression (Figure 8), we hypothesized that PRKCA might form a partnership and close correlation with PLCE1 along with the whole process of esophageal inflammation initiation, progression and tumorigenesis. Numerous studies have shown the association between PLCE1 and PI3K/AKT/mTOR signaling during inflammation-association carcinogenesis [44], thus targeting PI3K/AKT/mTOR-mediated inflammatory pathway could be a good strategy for cancer prevention and therapy [45]. Moreover, the expression levels, partnership or correlation of PLCE1 and PRKCA are also associated with ESCC patient outcome, for instance, low expression of both PLCE1 and PRKCA exhibited better survival of ESCC patients, but high expression of both PLCE1 and PRKCA showed the worst outcomes. Our recent work has reported that the stromal inflammation in esophageal squamous cell carcinoma occurs at the late stage and the inflammation is a immune response that protects the body from cancer cells, thus, ESCC stromal inflammation is associated with better outcomes [46]. However, esophagitis that occurs at early stage is a pre-cancerous lesion and facilitates carcinogenesis. With the advances in the management of gastrointestinal cancers [47], the combination of PLCE1 and PRKCA could be an immune checkpoint for esophageal cancer therapy.

## MATERIALS AND METHODS

### Human esophageal biopsies and esophageal cancer samples collection, quantitative RT-PCR and immunohistochemical staining

Human esophageal biopsies of 41 pairs of esophagitis and their adjacent normal esophageal mucosa were collected from the Central Hospital of Xinxiang Medical University (Xinxiang, China) and the Affiliated Hospital of Jining Medical University (Jining, China). The definition and classification of esophagitis has been reported recently by us [24]. The esophageal cancer tissues for tissue microarray (TMA) were collected from Linzhou Cancer Hospital, Linzhou, China. Total of 362 esophageal squamous cell carcinoma (ESCC) cases were put in a single TMA. All procedures were approved by Institutional Review Board and Ethic Committee of Xinxiang Medical University and Jining Medical University.

PLCE1 mRNA expression in esophageal biopsies was measured by quantitative RT-PCR (qRT-PCR) as we reported [24]. Similar procedure as analyzing PLCE1 was used to analyze PRKCA mRNA levels. Two sets of primers were listed in Supplementary Table 1. Anti-human PLCE1 Rabbit polyclonal antibody (Abcam, ab121477) and anti-human PRKCA monoclonal antibody (Abcam, ab32376) were used for immunohistochemical staining in the ESCC tissue microarray (TMAs). The stained slides were imaged using Aperio Image system. Diagnosis of esophagitis was made as we reported recently [24]. Immunohistochemical staining were scored as strong (scores 3 or 2) or weak (score 1) and no staining (score 0), similar as reported by us [24]. The staining scores were correlated with the clinicopathological characteristics and ESCC patients survival time using GraphPad Prism software.

### Oncomine data extract and analysis

Several sets of cancer data were downloaded from online cancer database Oncomine (https://www.oncomine.org). Two sets of Barrett's Esophagus and esophageal adenocarcinoma (EAC) data were extracted from Kim Esophagus [26] and Kimchi Esophagus [27], respectively; esophageal squamous cell carcinoma data was extracted from Hu Esophageal Cancer [25]; cervical cancer data was extracted from Zhai Cervix [30]; glioblastoma was extracted from Murat Brain Glioblastoma [31]; colorectal cancer data was extracted from Skrzypczak Colorectal Cancer [32]; hepatocellular carcinoma data was extracted from Roessler Liver Cancer [33]; renal cancer data was extracted from Jones Renal Cancer [34]; and yolk sac tumor data was extracted from Korkola Seminoma [35]; from which, the mRNA expression levels of PLCE1 and PRKCA were deeply mined and analyzed. GraphPad Prism software was used for statistical analysis. *p* < 0.05 was considered as statistical significance.

### N-nitrosomethylbenzylamine (NMBA)-treated rat esophagus and PLCE1 and PRKCA mRNA expression analysis

N-nitrosomethylbenzylamine (NMBA)-treated rat esophageal epithelial cells were scraped from formalin-fixed paraffin-embedded esophageal tissues that were provided by Dr,Li-Shu Wang (Medical College of Wisconsin, WI). As reported [48], male F344 rats (Harlan Sprague-Dawley, Indianapolis, IN, USA) were injected subcutaneously with 0.2 mL of 20% dimethyl sulfoxide (DMSO) in water (control) or NMBA (0.3 mg/kg of body weight) (Ash Stevens, Inc., Detroit, MI, USA) in 0.2 mL of NMBA vehicle three times per week for 5 weeks. The rats were sacrificed at 35 weeks by CO2 asphyxiation. The esophagus of each rat was opened longitudinally and fixed in 10% neutral buffered formalin. The tissues were embedded in paraffin and sectioned. Rat esophageal epithelial cells were scraped from the slides under microscope. Total RNA was extracted using Roche High Pure FFPE kit (Roche NimbleGen Inc, Madison, WI). PLCE1 and PRKCA mRNA was analyzed using qRT-PCR. The primers for rat PLCE1 and PRKCA were listed in Supplementary Table 1. All experimental protocols were in accordance with National Institutes of Health guidelines and approved by the Institutional Animal Care and Use Committee of University of Illinois at Chicago.

### PLCE1 knockout mouse esophagus and the analysis of PRKCA and cytokines

PLCE1 knockout (*PLCE1−/−*) and wild-type (*PLCE1+/+*) mouse esophagus was provided by Dr. Smrcka from the University of Rochester (Rochester, NY). The generation of the PLCE1 knockout mice has been reported [49]. For PLCE1 deficient mouse esophageal mucosa collection, it was briefed as following: PLCE1−/− and +/+ mice were sacrificed at 3 months, and the esophagus tissues were fixed in 70% ethanol, and then the esophageal mucosa was scraped under dissecting microscope using a scalpel. Total RNA was extracted and qRT-PCR was conducted to analyze the alterations of cytokines. The primers for cytokines TNFα, NF-kB, IL-1β, IFN-γ, IL-6, Cox2 and IKKβ have been reported by us [50] (also listed in Supplementary Table 1). The primers for mouse PRKCA and internal control GAPDH were listed in Supplementary Table 1. The Student's *t* test was used for qRT-PCR analysis. *p* < 0.05 indicated significant difference. All experimental protocols were in accordance with National Institutes of Health guidelines and approved by the Institutional Animal Care and Use Committee of University of Illinois at Chicago

### Cell culture and siRNA knockdown of PLCE1

Esophageal squamous cell carcinoma cell line EC9706 was purchased from Tumor Hospital Cell Culture Bank of Chinese Academy of Medical Sciences (Beijing, China). The cells were cultured and transfected with small interfering RNA targeting human PLCE1 (si-PLCE1) or scramble siRNA (NC, negative control), respectively. The cells were harvested at 48 h post transfection. Western blotting was performed to determine the expression of PLCE1 and PRKCA, qRT-PCR was performed to determine the expression of human cytokines. The sequences for human siRNA-PLCE1 and the primers for human cytokines were listed in Supplementary Table 1.

## CONCLUSIONS

The findings generated from this study have demonstrated that PLCE1 and PRKCA are well correlated in the development of esophageal inflammation and its malignant transformation, their correlation has critical clinical significance. This study has also strongly suggested that the co-expression of PLCE1 and PRKCA could be an indicator for predicting of esophageal cancer patient outcomes, and the partnership of PLCE1 and PRKCA could be a therapeutic target instead of a single target for personalized therapy or prevention.

## SUPPLEMENTARY MATERIALS TABLES



## Abbreviations

PLCE1: phospholipase c epsilon 1
PRKCA: protein kinase alpha
ESCC: esophageal squamous cell carcinoma
EAC: esophageal adenocarcinoma
