# Supplementary material for: Clinical significance of the correlation between PLCE 1 and PRKCA in esophageal inflammation and esophageal carcinoma
Source: Oncotarget. 2017 Mar 28;8(20):33285–99. doi: 10.18632/oncotarget.16635 (PMC5464868; doi:10.18632/oncotarget.16635)
Supplement: Supplementary file 1 [file oncotarget-08-33285-s001.pdf]

Clinical significance of the correlation between PLCE 1 and PRKCA in esophageal inflammation and esophageal carcinoma

Supplementary Materials

Supplementary Table 1: Sequences of the primers for qRT-PCR and for small interfering RNA

Primers for qRT-PCR analysis

Gene names	Forward-primer sequences	Reverse-primer sequences
hPLCε 1	TGTGGAACGAGCAGTTTCTG	ATCGAAGAGGC TGACATGGT
hPKCα	ATGTCACAGTACGAGATGCAAAA	GCTTTCATTCTTGGGATCAGGAA
hTNFα	ATGAGCACTGAAAGCATGATCC	GAGGGCTGATTAGAGAGAGGTC
hNF-kB	TCAATGGCTACACAGGACCA	ATCTTGAGCTCGGCAGTGTT
hIL-1β	GGGCCTCAAGGAAAAGAATC	TTCTGCTTGAGAGGTGCTGA
hIFN-γ	TCGGTAACTGACTTGAATGTCCA	TCGCTTCCCTGTTTTAGCTGC
hIL-6	ACTCACCTCTTCAGAACGAATTG	CCATCTTTGGAAGGTTCAGGTTG
hGAPDH	GTCAAGGCTGAGAACGGGAA	AAATGAGCCCCAGCCTTCTC
rPLCε1	GCACCAAAGCACACAGCTAA	GCTCCATCTCCTACCACCAA
rPKCα	TACAATGTGCCCATTCCAGA	CTTTCCAAAACTCCCCTTCC
r-actin-beta	CACCCGCGAGTACAACCTTC	CCCATACCCACCATCACACC
mPKCα	AGAGGTGCCATGAGTTCGTTA	GGCTTCCGTATGTGTGGATTTT
mTNFα	CCCTCACACTCAGATCATCTTCT	GCTACGACGTGGGCTACAG
mNF-kB	ATGTGCATCGGCAAGTCG	CAGAAGTTGAGTTTCGGGTAG
mIL-1β	GCAACTGTTCCTGAACTCAACT	GCAACTGTTCCTGAACTCAACT
mIFN-γ	GCCACGGCACAGTCATTGA	TGCTGATGGCCTGATTGTCTT
mIL-6	TAGTCCTTCCTACCCCAATTTCC	TTGGTCCTTAGCCACTCCTTC
mCOX-2	TGAGCAACTATTCCAAACCAGC	GCACGTAGTCTTCGATCACTATC
mIKKβ	CTGAAGATCGCCTGTAGCAAA	CTGAAGATCGCCTGTAGCAAA
mGAPDH	TCTGGAAAGCTGTGGCGTGAT	GCCAGTGAGCTTCCCGTTCAG

h: huamn; r: rat; m: mouse.

Small interfering RNA sequences for human PLCε 1 gene

Gene name-position	Sense (5′–3′)	Antisense(5′–3′)
PLCε 1-485	GCAUUAGUCCUUUAGGAAATT	UUUCCUAAAGGACUAAUGCTT
PLCε 1-2968	GCACCAAAGCACACAGCUATT	UAGCUGUGUGCUUUGGUGCTT
PLCε 1-4112	CCAGGUUUCUGAUGGAUAATT	UUAUCCAUCAGAAACCUGGTT
Scrambled siRNA	UUCUCCGAACGUGUCACGUTT	ACGUGACACGUUCGGAGAATT
